# N5 Is the New C4a: Biochemical Functionalization of Reduced Flavins at the N5 Position

**DOI:** 10.3389/fmolb.2020.598912

**Published:** 2020-10-30

**Authors:** Brett A. Beaupre, Graham R. Moran

**Affiliations:** Department of Chemistry and Biochemistry, Loyola University Chicago, Chicago, IL, United States

**Keywords:** flavin, covalent, N5-position, C4a-position, imino, oxide, peroxo

## Abstract

For three decades the C4a-position of reduced flavins was the known site for covalency within flavoenzymes. The reactivity of this position of the reduced isoalloxazine ring with the dioxygen ground-state triplet established the C4a as a site capable of one-electron chemistry. Within the last two decades new types of reduced flavin reactivity have been documented. These studies reveal that the N5 position is also a protean site of reactivity, that is capable of nucleophilic attack to form covalent bonds with substrates. In addition, though the precise mechanism of dioxygen reactivity is yet to be definitively demonstrated, it is clear that the N5 position is directly involved in substrate oxygenation in some enzymes. In this review we document the lineage of discoveries that identified five unique modes of N5 reactivity that collectively illustrate the versatility of this position of the reduced isoalloxazine ring.

## Introduction

**FIGURE 1:**
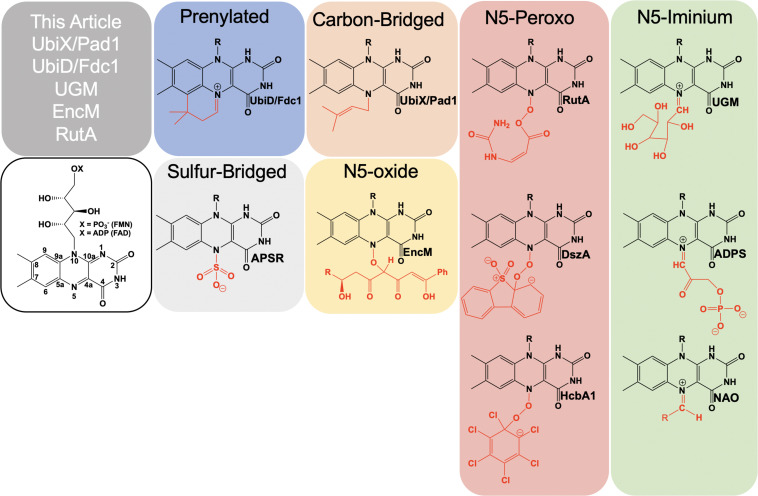
Examples of catalytically relevant flavin-N5 adducts.

Flavins are requisite to all life and are utilized as cofactors by enzymes throughout primary and secondary metabolism. They are typically enlisted by enzymes to facilitate oxidation/reduction reactions where the isoalloxazine ring system acts as a redox mediator between two other chemicals. Flavins are uniquely versatile in such reactions as the isoalloxazine can stabilize four oxidation states: four-electron oxidized, two-electron oxidized, one-electron oxidized and reduced. In addition to redox chemistries, flavins have been shown to facilitate reactions where no net redox change occurs (Sobrado, 2012). In such reactions, the reduced isoalloxazine ring acts as a nucleophile to generate covalent intermediates. Flavins extend their biochemical utility by covalent modification of the isoalloxazine either as an indelible modification or as a transient adduct (Figure 1). While covalent modifications of the C4a position have been known and studied for five decades, recently the versatility of the N5 position has come to the fore. Multiple lineages of flavin enzymes are now known to have evolved the capacity to form unique covalent modifications at the flavin N5 position that expand the known repertoire of flavin catalyzed reactions.

Formally, the most common functionalization of the flavin N5 position occurs with hydride transfer from a reductant substrate to form an N5-H adduct (reduced flavin). However, the ubiquity of this species in flavoprotein oxidases, dehydrogenases and other flavoenzymes and the fact that it is derived from the two-electron oxidized flavin precludes description in this article. Functionalization of an N5 by a moiety other than hydrogen was first described in 1967 when a photoalkylated C4a-lumiflavin adduct underwent isomerization (Walker et al., 1967). Soon after numerous examples of native and non-native N5 sulfite adducts with the oxidized isoalloxazine were described (Massey et al., 1969; Macheroux et al., 1999; Muller and Massey, 1969; Michaels et al., 1970; Mattevi et al., 1997a). N5 adducts formed in non-native reactions with flavo-enzymes are reported from 1970 onward (Michaels et al., 1970; Porter et al., 1973; Ghisla et al., 1976; Alston et al., 1983; Silverman et al., 1985; Mattevi et al., 1997b; Fraaije et al., 1998; Binda et al., 2001). However, it wasn’t until 1997 that the first verified example of a catalytically relevant N5 adduct forming with the reduced flavin of nitroalkane oxidase (NAO) was reported by Gadda et al. (1997). Soon after a native sulfate adduct was shown crystallographically in adenylylsulfate reductase (APSR) (Fritz et al., 2002). These discoveries solidified the notion of two primary adjacent catalytic centers in reduced flavin cofactors, C4a and N5.

In this article, we present recently identified flavo-enzymes that incorporate N5 modifications either during catalysis (intermediates) or as cofactor modification/maturation processes. This includes a pair isofunctional enzymes that form an N5-carbon adduct to catalyze the generation of a prenylated flavin mononucleotide (UbiX/Pad1) that is then utilized as a cofactor by prenylated flavin-dependent non-oxidative decarboxylase enzymes (UbiD/Fdc1). It also describes an example of a redox-neutral flavin reaction that uses the two-electron reduced flavin in catalysis and transiently forms an N5-galactose linkage (UDP-galactopyranose mutase; UGM). Lastly N5 oxo forms are presented; these utilize either the hyper-oxidized flavin N5-oxide (EncM) or the flavin N5-peroxide (RutA) to carry out oxygenation reactions.

## Prenylated: ubiX ubiD, pad1 and fdc1

**FIGURE 2:**
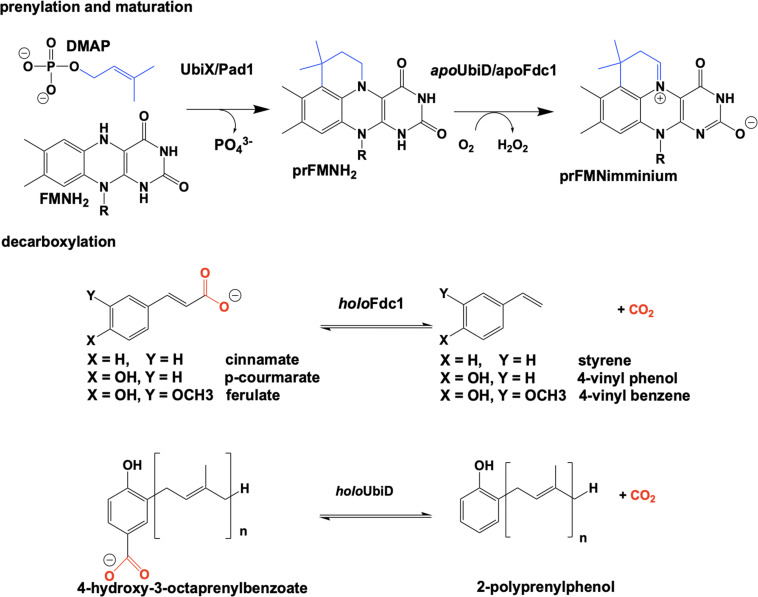
Overview of the proposed reaction of UbiX. UbiD, Pad1, and Fdc1.

The bacterial genes *ubiX* and *ubiD* and their respective fungal homologs *pad1* and *fdc1* encode for two pairs of enzymes associated with a reversible, non-oxidative decarboxylation of an aromatic substrate (Howlett and Bar-Tana, 1980; Cox et al., 1969; Leppik et al., 1976; Meganathan, 2001). These are essential steps in the biosynthesis of prokaryotic ubiquinone (Leppik et al., 1976; Howlett and Bar-Tana, 1980; Zhang and Javor, 2000; Gulmezian et al., 2007; Aussel et al., 2014) or fungal degradation of phenylacrylic/aromatic acids (Clausen et al., 1994; Stratford et al., 2007; Mukai et al., 2010; Richard et al., 2015). Due to similarities in the phenotype of gene deletion mutants, the proteins UbiX and UbiD, Pad1 and Fdc1 were initially believed to be isofunctional (despite minimal sequence similarity). Both sets of proteins where expressed together and thought to have redundant catalytic activity where bacterial UbiX and UbiD catalyze the decarboxylation of 3-octaprenyl-4-hydroxybenzoate to 2-octaprenylphenol (Zhang and Javor, 2003; Liu and Liu, 2006; Gulmezian et al., 2007; Jacewicz et al., 2013; Do et al., 2015; Richard et al., 2015) and eukaryotic Pad1 and Fdc1 catalyze the decarboxylation of cinnamic acid, coumaric acid and ferulic acid to styrene, 4-vinyl phenol and 4-vinyl benzene (Clausen et al., 1994; Larsson et al., 2001; Stratford et al., 2007; Richard et al., 2015). In 2015 it was determined that UbiX was in fact isofunctional with Pad1 and that neither possesses decarboxylase activity (Lin et al., 2015). Instead both function as prenyltransferases, catalyzing the formation of a prenylated four-ring flavin mononucleotide that is the cofactor for non-oxidative reversible decarboxylation reactions catalyzed by the UbiD and Fdc1 enzymes (Payne et al., 2015; White et al., 2015; Figure 2).

Reduced flavin mononucleotide (FMNH_2_) is prenylated by UbiX and Pad1. The N5-C6-prenylated flavin mononucleotide (prFMNH_2_) is bound as an immature cofactor by apoUbiD/apoFdc1 and then oxidized to the catalytically active prenylated flavin mononucleotide iminium ion (prFMN_iminium_) form (Figure 2). While decarboxylation reactions are not uncommon in the chemistries of living organisms and there are multiple examples of the enzymatic use of unmodified flavin for decarboxylation (Blaesse et al., 2000, 2003; Majer et al., 2002), the use of a prenylated FMN (White et al., 2015) for this chemistry was unprecedented.

The first structural insights into UbiX/Pad1 came with a 1.5 Å resolution crystal structure of UbiX from *Pseudomonas aeruginosa* (Kopec et al., 2011) (PDB CODE 3ZQU). It was observed that UbiX assembles into a dodecamer and binds FMN using Rossmann fold motif at the interface between three subunits. Crystal structures also revealed a small hydrophilic pocket adjacent to the *re*-face of the isoalloxazine moiety constructed of residues from the three proximal subunits and that neither face of the isoalloxazine was accessible to bulk solvent. Structural similarities to multiple enzymes related to decarboxylase activity (Blaesse et al., 2000, 2003; Kupke et al., 2001; Steinbacher et al., 2003; Manoj and Ealick, 2003) was one of the factors that initially prompted UbiX and Pad1 to be classified as FMN-dependent decarboxylase enzymes. However, no activity was seen when UbiX was incubated with substrate analogs 4-hydroxybenzoic acid, vanillic acid and 3-carboxymethyl amino-methyl-4-hydroxybenzoic acid and a classic redox role for FMN in the decarboxylation of benzoic acid derivatives (the apparent substrates of UbiD/UbiX) could not be established (Kopec et al., 2011).

In 2010 Mukai et al. examined a prior study (Ago, S., Kawasaki, H., and Kikuchi Y., Abst. 50th Annu. Meet. Soc. Biothechnol. p.24, 1998) that demonstrated saké yeast, normally devoid of ferulic acid decarboxylase activity due to lack of the *fdc1* gene, could exhibit resistance to ferulic acid when the gene *fdc1* (YDR539W) from wine yeast was incorporated into its genome indicating that Pad1/Fdc1 (UbiX/UbiD) are not isofunctional and that expression of both genes is required for decarboxylation activity. Confirmation came when it was demonstrated that cessation of decarboxylase activity in *Saccharomyces cerevisiae* occurred with the deletion of either the *fdc1* or *pad1* gene and that the resulting phenotype of either mutant was identical to the double mutant (Mukai et al., 2010).

**FIGURE 3:**
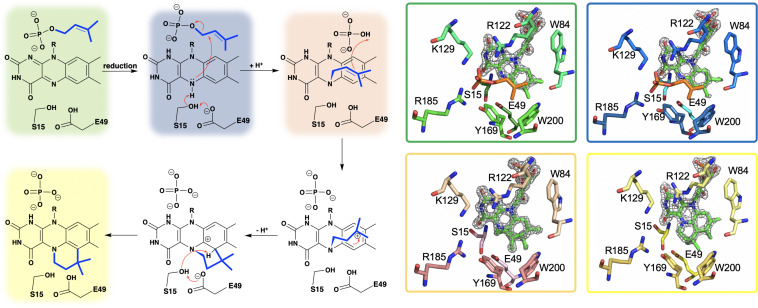
A hypothetical chemical mechanism for UbiX/Pad1 and supporting crystal structures of UbiX_ox_⋅DMP (dimethylallyl monophosphate) complex (PDB code 4ZAF, green), UbiX_red_⋅IMP (isopentyl monophosphate) complex (PDB code 6QLH, blue), UbiX-dimethylallyl monophosphate adduct (PDB code 4ZAV, tan), and UbiX⋅FMN_pren_ complex (PDB code 4ZAW, yellow).

Researchers from the University of Michigan proposed Pad1 and Fdc1 are part of a two component decarboxylase system where Pad1 activates Fdc1 and suggested it does so by providing Fdc1 with a covalently modified flavin mononucleotide cofactor (Lin et al., 2015). On this basis UbiX was incubated with oxidized FMN and dimethylallyl monophosphate (DMAP) and perturbation of the FMN UV-visible spectrum was observed (White et al., 2015), suggesting that DMAP binds in close proximity to the oxidized flavin. The binding of dimethylallyl monophosphate (DMAP) was confirmed by solving the crystal structure of the UbiX⋅FMN_ox_⋅DMAP complex (PDB CODE 4ZAF, 1.7 Å) (White et al., 2015) where DMAP binds adjacent to the *re*-face of the isoalloxazine moiety of the flavin monophosphate in a hydrophobic pocket reminiscent of that observed in terpene synthases and prenyl transferases (Doud et al., 2011; Lupoli et al., 2011; Gao et al., 2012; Figure 3).

The identity of the modified flavin mononucleotide produced by UbiX and Pad1 was determined when UbiX from *Pseudomonas aeruginosa* was incubated with reduced flavin mononucleotide (FMNH_2_) and DMAP resulting in the formation of a prenylated flavin mononucleotide (prFMNH_2_) (White et al., 2015; Figure 3). Crystals of UbiX could undergo turnover allowing researchers to use time-dependent crystallography matched with mass spectroscopy and/or UV visible spectroscopy to (1) confirm the binding of DMAP to the oxidized UbiX FMN (UbiX⋅FMN_ox_⋅DMAP) (PDB CODE 4ZAF), (2) show the structure of the active UbiX⋅FMNH_2_ complex (Glu49Gln variant UbiX⋅FMNH_2_⋅DMAP complex) (PDB CODE 4ZAL, 1.6 Å), (3) capture the initial N5-C1’ alkyl adduct (PDB CODE 4ZAV, 1.4 Å), (4) the product complex (UbiX⋅prFMNH_2_) (PDB CODE 4ZAW, 1.9 Å), and (5) observe the oxidation of the prFMNH_2_ to the purple oxygen dependent radical species (UbiX⋅prFMN_radical_) (PDB CODE 4ZAX, 1.6 Å) (Figure 3). Each of these structures formed sequentially when UbiX⋅FMN_ox_⋅DMAP was incubated with sodium dithionite (White et al., 2015). The structures in support of the proposed chemical mechanism in this sequence are shown in Figure 3 with a summary of the mechanism proposed from these structures. Active site mutations of UbiX revealed large conformational changes occur after reduction to yield species B that was then transformed to the stable N5 adduct C, before forming of the product, D (White et al., 2015).

Ubix/Pad1 offers the first example of a flavin monophosphate dependent prenyl-transferase and forms a new flavin cofactor, an bridged N5 and C6 dimethyl prenylflavin. The enzymes UbiX and Fdc1 ensure a stable N5-alkyl adduct flavin species (prFMNH_2_) is favored over the reactive N5-iminium adduct (prFMN_iminium_) by utilizing the reduced from of the flavin monophosphate to act as a nucleophile. This strategy dictates that the reduced prenylated flavin monophosphate product of UbiX/Pad1 is transferred to apo-UbiD/Fdc1 before undergoing activation/oxidation to the reactive imine form.

When the *fdc1* gene from *Aspergillus niger* and the gene *ubiX* from *E. coli* were coexpressed in *E. coli*, the resulting decarboxylase enzyme (Fdc1_UbiX_) displayed distinct spectral properties when compared to singly expressed Fdc1. Fdc1_UbiX_ catalyzed the decarboxylation of multiple aromatic carboxylic acids *in vitro* (Payne et al., 2015), providing the first definitive evidence of dependent catalytic activity and the first indication that UbiD/Fdc1 are exclusively responsible for decarboxylation. The crystal structures of Fdc1_UbiX_ using Fdc1 from *A. niger* (PDB CODE 4ZA4, 1.2 Å), *Candida dubliniensis* (PDB CODE 4ZAD, 2.5 Å) and *Saccharomyces cerevisiae* (PDB CODE 4ZAC, 1.7 Å) confirmed the identity of the prenylated flavin mononucleotide (Payne et al., 2015). The crystal structures also revealed the presence of a metal ions in complex with the ribityl phosphate of the prenylated FMN, one of which was confirmed to be Mn^2+^ by EPR and the other is thought to be K^+^ (Payne et al., 2015; Bailey et al., 2018), consistent with early reports of the dependence of UbiX/Pad1 and UbiD/Fdc1 on Mn^2+^ (Leppik et al., 1976).

The first crystal structure of UbiD from *E. coli* (PDB CODE 2IDB, 2.9 Å) was solved in 2006 by the Northeastern Structural Genomics Consortium and revealed a hexameric quaternary structure. However, due to the limited resolution of the data, specific details of residue identity and position could not be established. In 2013, a 1.9 Å-resolution crystal structure of the dimeric UbiD from *Pseudomonas aeruginosa* (PDB CODE 4IP2) was determined and the associated electron density maps displayed clear difference electron density indicative of a Mg^2+^ metal center (Jacewicz et al., 2013). A large cleft on the face of the structure extended from the metal binding site across 2 domains and terminated near a pocket of conserved residues Arg170, Glu273, and Glu278 that was designated as the putative active site. It was also noted that residues Glu229 and His188 from UbiD were structurally conserved with Glu105 and His68 in FMN-binding protein from *Methanobacterium thermoatuotrophicum* (Christendat et al., 2000) (PDB CODE 1EJE, 2.2 Å). These residues are believed to be involved with the binding of flavin mononucleotide whose phosphate was bound to a similarly positioned metal ion. However, binding of FMN to UbiD could not be demonstrated by conventional methods (Jacewicz et al., 2013).

**FIGURE 4:**
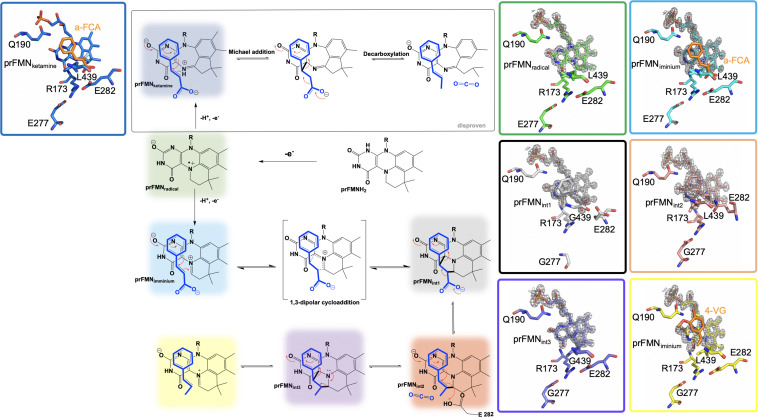
Proposed catalytic mechanism of Fdc1_UbiX_ and supporting crystal structures of prFMN_ketamine_⋅α-FCA (α-fluoro cinnamic acid) (PDB code 4ZAB, blue), prFMNradical (PDB code 6R2T, green), prFMNiminium⋅α-FCA complex (PDB code 4ZAB, light blue), prFMN_int1_ adduct (PDB code 6R3O, black), prFMN_int2_ adduct (PDB code 6R3F, tan), prFMN_int3_ adduct (PDB code 6R3J, purple), and prFMNiminium⋅4-VG (4-vinyl glycol) complex (PDB code 4ZAA, yellow).

Crystals of Fdc1_UbiX_ from *A. niger* were soaked with various *trans-*cinnamic acid-related compounds: 4-vinyl guaiacol (PDB CODE 4ZAA, 1.2 Å), penta-fluorocinnamic acid (PDB CODE 4ZA8, 1.1 Å), phenylpyruvate (PDB CODE 4ZA9, 1.0 Å) and alpha-fluoro cinnamic acid (PDB CODE 4ZAB, 1.2 Å) (Payne et al., 2015). The structures of these complexes indicate that the substrate’s enoic acid double bond stacks directly above the oxidized prenylated flavin (prFMN) cofactor that was observed in two configurations, an isoalloxazine N5-iminium (prFMN_iminium_) and an N5-ketamine species (prFMN_ketamine_) (Payne et al., 2015; Figure 4).

UbiD and Fdc1 bind prenylated flavin mononucleotide in the reduced form (prFMNH_2_) which then requires activation by oxidation to yield the iminium (prFMN_iminium_) or possibly the ketamine (prFMN_ketamine_) form (Payne et al., 2015; Figure 4). Conceivably, acid/base chemistry could utilize the N5 of the secondary ketamine as a catalyst, taking advantage of the positioning of the substrate’s α,β-unsaturated carbonyl above the prFMN_ketamine_-C4a to undergo Michael addition-like chemistry that has been observed in other flavin containing enzymes (Koehn and Kohen, 2010; Walsh and Wencewicz, 2013), forming a transient C4a-substrate adduct prior to decarboxylation. The alternative would be to utilize prFMN_iminium_ where the substrate α,β-unsaturated carbonyl, a dipolarophile, interacts with the 1,3-dipole arrangement of prFMN_iminium_ (Huisgen, 1963), to undergo what would be the first biological example of a 1,3 dipolar cycloaddition leading to the formation of a substrate-prFMN_iminium_ adduct bridging both the C4a and C1’ of the prFMN_iminium_ cofactor (Figure 4). The bridged substrate-prFMN_iminium_ could then undergo a Grob-like decarboxylation (Prantz and Mulzer, 2010) of the pyrrolidine adduct that could be coupled to cleavage of the C4a-substrate bond resulting in a prFMN-C1’-substrate adduct where protonation by Glu282 leads to the formation of the second double-bridged pyrrolidine adduct. This adduct would then be subject to retro 1,3-dipolar cycloaddition resulting in the formation of the product complex (Figure 4).

In an attempt to determine which pathway is utilized, Fdc1_UbiX_ from *A. niger* was incubated with phenylpyruvate resulting in reversible inhibition of the enzyme by the enol tautomer. Crystal structures of this complex revealed the phenylacetaldehyde adduct with what would have been the imino form of prFMN. Moreover, crystallization in the presence of α-hydroxycinnamic acid indicated an α-hydroxystyrene prFMN-adduct, both confirming prFMN_iminium_ as the active form of the enzyme (Payne et al., 2015). Additional crystal structures of Fdc1 were solved in 2018 and 2019 and provided additional physical evidence in support of this mechanism in the form of a structure of Fdc1prenylated FMN in radical form and substrate analog phenylpropiolic acid (PDB code 6R2T, 1.3 Å) (Bailey et al., 2019; Figure 4). Crystal structures representative of prFMN_int__1_ and prFMN_int__2_ were solved using L439G variant Fdc1 in the presence of phenylpropiolic acid (PDB code 6R30, 1.1 Å) and variant E282Q in the presence of cinnamic acid (PBD code 6R3F, 1.3 Å), respectively (Bailey et al., 2019; Figure 4). Characterization of intermediate species was possible due to active site variant Fdc1, where L439G mutation resulted in a decrease in *k*_cat_ and a substantial increase in K_M_ and as a result the reaction ceased to undergo decarboxylation and confirmed the role of Phe437 in catalysis. Mutation of the glutamic acid responsible for the protonation (Bailey et al., 2018) and E282Q abolishes catalysis with cinnamic acid and resulted in the formation of a distinct species determined to be prFMN_int__2_ (Bailey et al., 2019; Figure 4). Evidence for the subsequent species, prFMN_int__3_, was obtained from WT Fdc1 crystal structures that were flash-cooled proceeding exposure to cinnamic acid (Bailey et al., 2019) (PDB code 6R3J) and revealed a prFMN crotonic acid adduct predicted to form after decarboxylation and protonation (Figure 4). The size and diverse nature of the crystal structure catalog and the formation of multiple dead-end complexes afforded from reactions with substrate analogs and/or variant forms of Fdc1 provide a composite of support for decarboxylation coupled to reversible 1,3 dipolar cycloaddition.

Kinetic insight into the mechanistic progression of Fdc1_UbiX_ from *S. cerevisiae* where solvent and secondary deuterium isotope effects at the α and β positions of the phenylacrylic acid substrate support a mechanism involving 1,3 cycloaddition (Ferguson et al., 2016). These observations indicate that carbon dioxide release is dependent on proton transfer to the product where proton transfer must precede the rate limiting step. The identification of the rate limiting step was determined from a linear free-energy analysis using a range of *para* and *meta*-substituted phenylacrylic acids where Hammett parameters were consistent with formation of styrene via cyclo-elimination as rate limiting (Ferguson et al., 2016). Additionally, reactions carried out in the presence of mechanism-based inhibitor 2-fluoro-2-nitro-vinylbenzene terminated with a cyclo-addition adduct and provide direct evidence that prFMN based chemistry proceeds via 1,3-dipolar cyclo-addition (Ferguson et al., 2017).

## Covalent N5-Iminium: UDP-Galatopyranose Mutase

**FIGURE 5:**
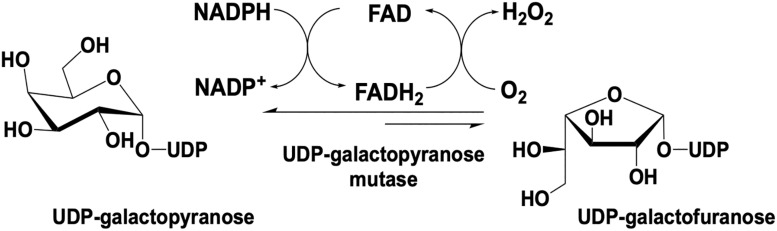
Reaction catalyzed by UDP-galactopyranose mutase.

UDP galactopyranose mutase (UGM) is a flavin dependent protein that catalyzes the 6- to 5-member sugar ring contraction of UDP-galactopyranose (UDP-galp) to form UDP-galactofuranose (UDP-galf). The reaction catalyzed transiently forms a covalent FAD-galp iminium complex intermediate necessary for nucleophilic attack and ring closure (Figure 5). This reaction has an equilibrium position that favors UDP-galp (11:1) as a result of the increased ring strain generated in the contracted saccharide (Nassau et al., 1996). UGM is active in the reduced state of the flavin, the reaction is overall redox neutral and bifunctional in that it can recover the FMNH_2_ after oxidation by acquiring electrons from NAD(P)H.

UDP-galf is a required precursor for the biosynthesis of galactofuranose-dependent glycoconjugates found in bacteria, fungi, and protists (Koplin et al., 1997; Weston et al., 1997; Beverley et al., 2005; Novelli et al., 2009). In mycobacteria species, UGM is responsible for the biosynthesis of precursors used for glycoconjugates that function in anchoring the outer mycolic acid layer to the peptidoglycan of the cell wall. Mutants of *Mycobacterium smegmatis* that lack the gene *glf*, which codes for UGM, lacked the ability to grow at non-permissive temperatures confirming that UGM is essential for infective growth in these organisms (Pan et al., 2001). UGM was also shown to have importance in the fungus *Aspergillus fumigatus*, the causative agent of aspergillosis that poses a lethal pulmonary threat to immunocompromised individuals (Latge, 1999). Deletion of *glf* in *A. fumigatus* resulted in attenuated virulence in a low dose mouse model that was attributed to a decrease in the thickness of the cell wall resulting in an increased susceptibility to antifungal agents (Schmalhorst et al., 2008). In kinetoplastids such as the protozoan species *Leishmania major* the monosaccharide product of UGM serves as a precursor for galactofuranose, which anchors lipophosphoglycans and glycoinositolphospholipids to the cytosolic surface of the cell (Turco and Descoteaux, 1992). Mutant *L. major* devoid of UGM activity paralleled growth of wild type species *in vitro* but displayed attenuated virulence (Kleczka et al., 2007). These observations have spurred scientific investigation into the catalytic and structural characteristics of UGM in these organisms; this activity is absent in higher organisms, making UGM a target for anti-microbial, anti-fungal and anti-parasitic agents (Pan et al., 2001; Richards and Lowary, 2009). As such, several recent studies have presented UGM inhibitor candidates with micromolar dissociation constants (Soltero-Higgin et al., 2004b; Veerapen et al., 2004; Itoh et al., 2007; Dykhuizen et al., 2008; Borrelli et al., 2010; Partha et al., 2010; El Bkassiny et al., 2014; Kuppala et al., 2015; Mahdavi-Amiri et al., 2016; Kashif et al., 2018).

Initial reports indicated that UGM is FAD-dependent (Nassau et al., 1996) and earlier reports had indicated that the oxidized form of the flavin is required for activity (Zhang and Liu, 2001). However, it was later determined that UGM is only active when the flavin is in the reduced form (Zhang and Liu, 2000; Sanders et al., 2001; Goni et al., 2008; Oppenheimer et al., 2010, 2011; Maaliki et al., 2020). Eukaryotic UGMs from *A. fumigatus* and the protozoa *T. cruzi*, *L. Mexicana*, and *L. infantum* utilize NADPH as a co-substrate and can both reinstate and stabilize the reduced form of the enzyme under aerobic conditions (Dhatwalia et al., 2012c; Oppenheimer et al., 2012; Fonseca et al., 2013). It was also demonstrated that once reduced, eukaryotic UGMs can complete an average of several hundred turnovers before oxidation/inactivation occurs. This behavior occurs as a result of the rate of oxidation occurring 200 to 1500-fold slower than rate of re-reduction (Dhatwalia et al., 2012c; Fonseca et al., 2013; Tanner et al., 2014). Bacterial UGM can also utilize NADPH for enzyme reactivation but requires a large excess of NADPH and extended incubation times due to a 10,000-fold slower reduction rate compared to eukaryotic UGM, suggesting that alternative physiological reductants may be utilized by the bacterial forms *in vivo* (Barlow et al., 1999; Gusarov et al., 2008). Kinetic analysis demonstrated that UGM from *T. cruzi* and *A. fumigatus* undergo reduction of the flavin cofactor by NADH, 7- and 17-fold slower, respectively, than by NADPH but exhibit dissociation constants for NADH that are 5- and 10-fold lower than those for NADPH indicating no distinct preference in terms of k_red_/K_d_ (Tanner et al., 2014).

**FIGURE 6:**
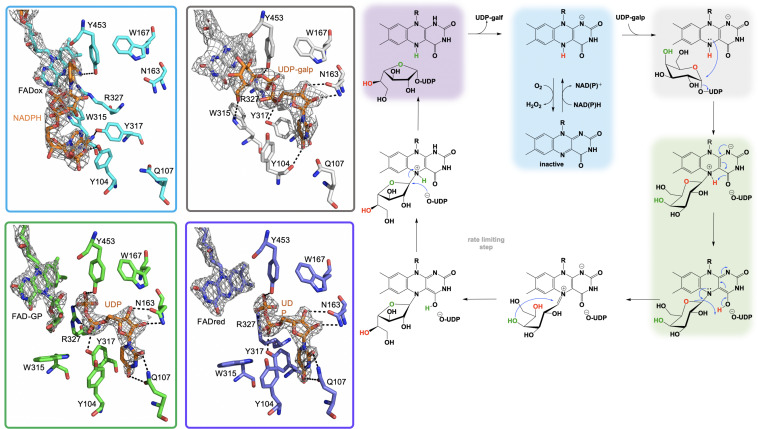
The active site substrate contacts observed in the structure of the *A. fumigatus*, UGMox⋅NADPH complex (PBD Code 4VWT, light blue) UGMox⋅UDP-galp complex (PDB Code 3UKH, gray), H63A variant UGMox-galpoUDP complex (PDB Code 5HHF, green), and UGMredoUDP complex (PDB Code 3UTG, purple).

The most widely accepted chemical mechanism for UGM utilizes the flavin as a nucleophile to facilitate an attack on the anomeric carbon of the substrate displacing (but retaining) UDP. In this mechanism the binding of the substrate (UDP-galp) to reduced UGM results in a local reorganization of the active site resulting in positioning of the N5 of the FAD proximal (3.4 Å) to the C1-galp (Gruber et al., 2009b; Partha et al., 2009; Dhatwalia et al., 2012c; van Straaten et al., 2012a,b, 2015). This conformation facilitates a direct attack of the substrate anomeric carbon by the reduced flavin in a proposed S_N_2-like mechanism (Sun et al., 2012; Figure 6). Direct attack by the flavin was also supported by studies where substitution of FAD with 5-deaza-FAD resulted in inactive enzyme (Huang et al., 2003). The results of 5-deaza-FAD substitution studies were initially proposed to be evidence in support of a mechanism involving single electron transfer (Huang et al., 2003), however, the linear free energy relationship of k_cat_ to the nucleophilicity of the N5 position of UGM substituted with various flavin analogs resulted in a ρ value of −2.4, consistent only with a direct nucleophilic attack mechanism (Soltero-Higgin et al., 2004a).

It is proposed that the nucleophilic attack of the FAD N5 of the reduced flavin UGM on the C1 of UDP-galp results in the formation of a covalent FAD-sugar adduct with the displacement of the UDP (Figure 6, green). The formation of a covalent FAD reaction intermediate was confirmed in eukaryotic UGMs by UV/vis spectrophotometry and mass spectrometry (Oppenheimer et al., 2012). The intermediacy of an N5-covalent adduct in the UGM mechanism was confirmed by Kiessling and coworkers using borohydride to reduce the putative iminium state and form an N5-alkyl flavin which was also identified by UV/vis spectrophotometry and LC/MS (Soltero-Higgin et al., 2004a). The covalent FAD-galactose intermediate was also confirmed in prokaryotic UGMs by mass spectrometry, NMR and most recently by an X-ray crystal structure of *A. fumigatus (variant H63A)* (PDB CODE: 5HHF) (Figure 6, green) (Gruber et al., 2009a,b; Mehra-Chaudhary et al., 2016). It was suggested that the sugar-FAD adduct would facilitate ring opening and activation of the C1 of galp where the flavin would act as a structural scaffold providing the dimensional constraints required for ring contraction (Soltero-Higgin et al., 2004a; Sobrado, 2012; Tanner et al., 2014; Pierdominici-Sottile et al., 2018). This mechanistic progression is also consistent with positional isotope exchange (PIX) studies that determined that the glycosidic bond of UDP-galp is cleaved during catalysis (Barlow et al., 1999; Zhang and Liu, 2001). The exact identity of the captured covalent sugar adduct in crystal structures from *A. fumigatus* could not be identified, however, researchers concluded it must be one of the two species depicted in green in Figure 6 (Mehra-Chaudhary et al., 2016).

Kinetic studies conducted with UGM from *Trypanosoma cruzi* showed no evidence of the formation of a flavin semiquinone species during turnover (Dhatwalia et al., 2012b) despite earlier reports suggesting the flavin semiquinone is stabilized by substrate binding (Fullerton et al., 2003). Interestingly, these kinetic studies did reveal the formation of a possible flavin iminium ion intermediate (Figure 6; Oppenheimer et al., 2012). Hybrid quantum/classical calculations suggest that C4 = O of the FAD accepts a proton from N5 of the FAD and subsequently donates it to the O5 of Galp (Huang and Gauld, 2012). FAD iminium ion formation would require deprotonation of the FAD N5 of the covalent FAD-sugar adduct, thereby facilitating opening of the ring of galp between C1 and the ether oxygen (Tanner et al., 2014). Hybrid simulations also predict that the proton for the C4-OH of Galp is shuttled to the C4 = O of FAD during the contraction of the ring resulting in a covalent FAD-galf adduct (Huang and Gauld, 2012). To date there is no biochemical information on the mechanisms of cleavage of the covalent FAD-galf complex or reconstruction of the galf-UDP molecule or the order in which they occur.

Kinetic analysis of UGM from *T. cruzi* demonstrated that the formation of the FAD iminium species occurs much more rapidly (300 s^–1^) than the observed rate of catalysis (k_cat_ ∼ 10 s^–1^) and viscosity effects studies have ruled out product release as the rate-limiting step (Oppenheimer et al., 2012; Tanner et al., 2014). On this basis, it was proposed that the rate of catalysis is governed by either the ring contraction or reattachment of UDP (Oppenheimer et al., 2012). Additionally, studies on the rate limiting step in UGM from *A. fumigatus* recorded kinetic isotope effects of ∼2 during the conversion of UPD-galp/galf and solvent viscosity effects suggesting that slow proton transfer dictates the rate limiting step and that product release is only partially rate limiting (Pierdominici-Sottile et al., 2018). This study also determined that structural recognition of the hydroxymethyl group of the hexose C5 provides a kinetic barrier to cyclization and mutation of tryptophan 315 to alanine results in a 370-fold decrease in *k_cat_/K*_M_ compared to wildtype UGM where in the variant product release describes k_cat_ and that effect could be mimicked in wildtype UGM when utilizing UDP-arabinopyranose as a substrate (Pierdominici-Sottile et al., 2018).

The three-dimensional structure of UGM has been extensively characterized and revealed a complex topology consisting of three domains (Sanders et al., 2001). The structure of domain one is non-contiguous containing an abbreviated Rossmann fold responsible for FAD binding, however, UGM from *E. coli* lacks the terminal strand of the Rossmann fold (Cristescu and Egbosimba, 2009). Domain 2 is contiguous and includes a cluster of five α-helices that form the binding site for the uridine group, the active site “flap 1” (residues 167 to 177 in *K. pneumoniae* UGM) that undergoes UDP-galp dependent conformational changes and has a role in dimerization (Beis et al., 2005). The third domain is non-contiguous and consists of a 6-stranded antiparallel β-sheet. The structures of all bacterial UGMs are topologically similar where secondary-structure matching analysis revealed that 73–96% of secondary structure elements found in UGM from *E. coli* are completely conserved across all other known bacterial UGMs (Krissinel and Henrick, 2004) despite substantial variation in primary structure (37–44% pairwise identity with UGM from *E. coli*) (Tanner et al., 2014).

In all, 59 structures of UGM from 9 different species including prokaryotic strains from; *Campylobacter jejuni, Corynebacterium diphtheriae, Deinococcus radiodurans, Mycolicibacterium smegmatis, Klebsiella pneumoniae, Mycobacterium tuberculosis*, and *Escherichia coli* as well as examples from the eukaryotes *A. fumigatus* and *T. cruzi* have been solved (Sanders et al., 2001; Beis et al., 2005; Gruber et al., 2009a,b; Partha et al., 2009, 2010; Dhatwalia et al., 2012a,b,c; van Straaten et al., 2012b, 2015; Da Fonseca et al., 2014; Poulin et al., 2014; Tanner et al., 2014; Kincaid et al., 2015). These structures included examples of UGM in both the oxidized and reduced states and in complex with multiple ligands including NADH, NADPH, flavin mononucleotide (FMN), UDP-phosphono-galactopyranose, UDP-galactose, UDP, UDP-galp and dideoxy-tetrafluorinated analogs, both the substrate and product, UDP-F_4_-galp and UDP-F_4_-galf as well as the potential inhibitor 2-[4-(4-chlorophenyl)-7-(2-thienyl)-2-thia-5,6,8,9-tetrabicyclo[4.3.0]nona-4,7,9-trien-3-yl]acetate (Beis et al., 2005; Gruber et al., 2009a,b; Partha et al., 2009, 2010; Dhatwalia et al., 2012a,b; van Straaten et al., 2012a,b). Single site directed variant UGM structures of Phe66Ala, Tyr317Ala, Asn207Ala, Gln107Ala, Tyr104Ala, and His63Ala variants of the *A. fumigatus* UGM have also been determined (Da Fonseca et al., 2014; Tanner et al., 2014; Kincaid et al., 2015; van Straaten et al., 2015). Possibly the most directly edifying structures with regard to the UDP-galp chemistry are the UGM_red_⋅NADP^+^ (PDB code 4VWT, 2.8 Å), UGM_red_⋅UDP (PDB code 3UTG, 2.3 Å), UGM_ox_⋅UDP-galp (PDB code 3UKH, 2.3 Å) and the covalent UGM_ox_-galp⋅UDP (PDB code 5HHF, 2.3 Å) crystal structure complexes depicted in Figure 6.

Multiple challenges in structure determination were encountered for UGM from eukaryotic species, *A. fumigatus* (Dhatwalia et al., 2012a; Penman et al., 2012; van Straaten et al., 2012a) and *L. major* (van Straaten et al., 2012b), including translational pseudo-symmetry and crystal twinning. It was ultimately determined that mutagenesis of long-chain charged surface residues, specifically lysine and glutamine allowed for reproducible growth of crystals that were free of crystallographic pathologies (Derewenda, 2004; Goldschmidt et al., 2007; Dhatwalia et al., 2012a). Two independent groups solved the variant crystal structures of UGM from *A. fumigatus* using either the double variant Lys344Ala/Lys345Ala (Dhatwalia et al., 2012a) or the single variant Arg327Ala (van Straaten et al., 2012a) and the two structures produced are almost identical. These structures revealed that eukaryotic UGM consists of three domains similar to UGMs from prokaryotes with noticeable variations. Domain one retains the shortened Rossmann-fold described for prokaryotic UGMs but has an additional 4-stranded antiparallel β-sheet that sits adjacent to the Rossmann fold and an extended ∼30 residue C-terminus that plays a role in the tetrameric assembly of UGM from *A. fumigatus*. Domain two consists of a bundle of α-helices similar to prokaryotic enzymes but contains an additional α-helix and a helical extension (∼7 residues), which are involved in tetramer formation. The additional α-helix forms the scaffold for a mobile active site “flap 2” absent in prokaryotic UGMs. Domain three contains a twisted, seven-stranded antiparallel β-sheet compared to the six-stranded β-sheet seen in prokaryotes. More recently the crystal structure of UGM from *T. cruzi* was solved in both oxidized (PDB code 4DSG) and reduced (PDB code 4DSH) states (Dhatwalia et al., 2012b). The protomer structure was highly similar to *A. fumigatus* (RMSD 1.1 Å) (Tanner et al., 2014), though UGM from *T. cruzi* crystallized as a monomer as opposed to the homotetramer, consistent with results of small angle X-ray scattering (Dhatwalia et al., 2012b).

Additionally, crystal structures of eukaryotic UGM from *A. fumigatus* were solved in complex with NADH (2.8 Å, PDB code 4GDD) and NADPH (2.8 Å, PDB code 4GDC) and have provided a valuable description of the unique NAD(P)H binding site (Dhatwalia et al., 2012c). The NAD(P)H molecule is sandwiched between domain one and three, forming interactions with both and is positioned in an unusually constrained conformation that positions the adenine moiety within hydrogen bonding distance of the ribose hydroxyl (Da Fonseca et al., 2014), an arrangement that is wholly different than the Rossmann dinucleotide-binding fold described for most enzymes that utilize NAD(P)H as a substrate (Bottoms et al., 2002; Kleiger and Eisenberg, 2002). Multiple conserved residues (Asn57, Ile65, Phe66, His68, Arg91, Ser93, Tyr104, and Tyr317) are involved in stabilizing the ENAD(P)H complex where hydrogen bonding between Tyr104 and the 2’ phosphoryl group of NADPH is responsible for the ∼180-fold preference (k_cat_/K_D_) for NADPH over NADH seen in UGM from *A. fumigatus* (Dhatwalia et al., 2012a). However, binding of NAD(P)H molecules does not induce conformational changes in either of the two dynamic active site flaps (residues 179 to 187 and residues 203 to 209) responsible for access to the active site (Dhatwalia et al., 2012a). Interestingly, the bound NAD(P)H molecule extends into the UPD-galp binding pocket and implies that binding of NAD(P)H and UDP-galp are mutually exclusive (Tanner et al., 2014). Prokaryotic UGM lacks all of the conserved residues that contact the ADPP moiety of NADPH seen in eukaryotic UGM indicating that prokaryotic UGM does not contain a recognizable NADPH binding domain which is consistent with biochemical data (Tanner et al., 2014).

## EncM

**FIGURE 7:**
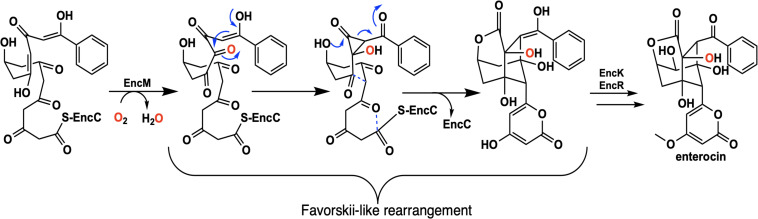
The reaction catalyzed by EncM and subsequent conversion to enterocin by EncK and EncR.

Flavins have been regarded as participating in a well-defined set of reaction categories (Hemmerich et al., 1970; Mansoorabadi et al., 2007; Chaiyen et al., 2012; Sobrado, 2012; Wencewicz and Walsh, 2012). Flavin-dependent monooxygenases were thought to almost exclusively stabilize a C4a-(hydro)peroxyflavin intermediate as the oxygenating entity (Massey, 1994; De Colibus and Mattevi, 2006; Palfey and McDonald, 2010; Crozier-Reabe and Moran, 2012; Wencewicz and Walsh, 2012). In 2013, a unique bacterial enzyme, EncM, was described. This enzyme catalyzed the C4a-peroxyflavin independent oxygenation and dehydrogenation of a poly(β-carbonyl) substrate, promoting a Favorskii-like rearrangement to form desmethyl-5-deoxyenterocin which is then further transformed by enzymes EncK and EncR to form the antibacterial enterocin (Figure 7). EncM was ultimately found to utilize a covalent flavin N5-oxide as the oxygenating species (Teufel et al., 2013, 2015). This highly unusual species is the first example of a four-electron oxidized flavin cofactor (Teufel et al., 2013).

**FIGURE 8:**
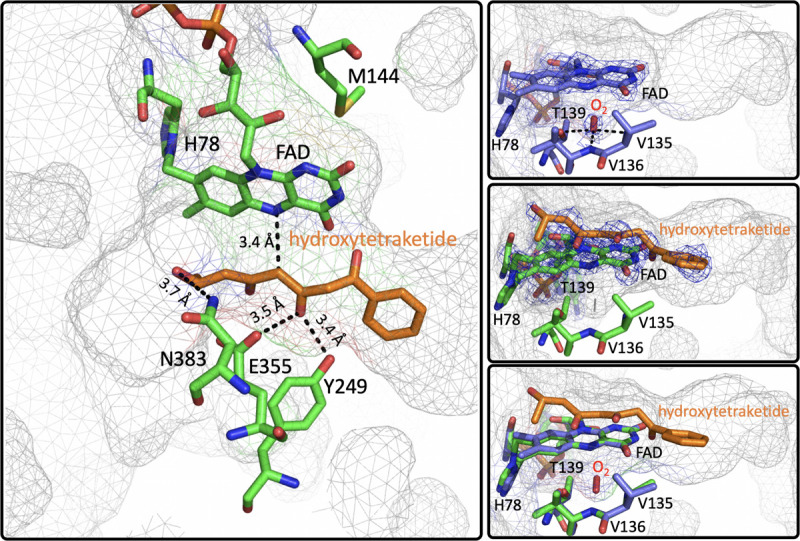
The Active Site of EncM. Left. EncM with a hydroxytriketide substrate analog bound (PDB code 3W8Z). Right top, EncM crystalized in the presence of 15 bar dioxygen (PDB code 6FOQ). Right middle, EncM in complex with a hydroxytriketide substrate analog bound (PDB code 3W8Z). Right bottom, overlay of both structures. The mesh indicates the shape of the terminal end of the internal cavity. Electron density is shown in blue and derived from 2fo-fc maps.

EncM functions in the biosynthesis of enterocin in multiple streptomycete bacteria (Piel et al., 2000; Hertweck et al., 2004; Xiang et al., 2004; Cheng et al., 2007). Dual oxidation reactions of the otherwise promiscuously reactive poly(β-carbonyl) substrate promote the Favorskii-type rearrangement resulting in the formation of the cyclohexanyl and lactam rings of enterocin (Seto et al., 1976; Teufel et al., 2013). The first crystal structure of unliganded EncM was published in 2013 (PDB code 3W8W, 2.0 Å) along with a structure determined in the presence of NADPH (PDB code 4XLO, 1.7 Å, *vide infra*). These structures revealed that, consistent with quaternary structure determinations, EncM crystallizes as a homodimer (Teufel et al., 2013). The structures also showed that residues 2–210 and 419–461 form an FAD-binding domain where covalent binding of the C8-methyl of the isoalloxazine moiety to His78, positions the flavin isoalloxazine moiety adjacent to the substrate-binding domain where it forms one internal face of the active site (Figure 8). The active site is adjacent to a group of positively charged residues at the protein’s surface that form a basic region that is complementary in charge and shape to the negative surface of the EncC carrier protein (Figure 7; Xiang et al., 2004; Crosby and Crump, 2012).

On this basis it was proposed that this electrostatic surface complementarity enhances protein-protein interaction between the polyketide carrier protein, EncC and EncM, limiting deleterious side reactivity of the substrate poly(β-carbonyl) chain (Piel et al., 2000; Teufel et al., 2013). Additionally, mutagenesis studies designed to disrupt this protein-protein interface resulted in a ∼40% decrease in activity compared to wild type (Teufel et al., 2013). Additional crystal structures of EncM were solved in complex with the substrate analogs: trifluorotriketide (PDB code 3W8X, 1.8 Å) and hydroxytetraketide (PDB code 3W8Z, 1.8 Å) (Teufel et al., 2013; Figure 8). These structures indicate that the terminal benzene group forms multiple van der Waals and π-stacking interactions with hydrophobic residues of EncM. These interactions position the enol proximal to the C1 of the substrate analog(s) within hydrogen bonding distance (2.4 Å) of the O(4) of the flavin. The key active site residues lie on the terminal limb of an L-shaped tunnel (~30 Å in length that extends to the surface of the enzyme near the dimer interface. The shape of this tunnel is complementary to an open conformation of the acyl carrier protein bonded phosphopantetheine arm and the ketide chain of the 1,3-diketone substrate, whose presumed function is to physically separate the phenyl-tetraketide head from the remaining tetraketide tail and suppress adventitious cyclization/aromatization reactions (Teufel et al., 2013).

Interestingly, the active form of EncM has a spectrum that closely resembles that of two-electron oxidized flavin. The enzyme is observed to inactivate after an average of only seven turnovers and this results in a blue shift and an increase in the extinction coefficient of the isoalloxazine absorption transitions (Teufel et al., 2015). It was proposed that inactivation resulted from the loss of the flavin oxygenating species (Teufel et al., 2013, 2015). Reactivation was observed in the presence of NAD(P)H and dioxygen. While, EncM does not have a catalytic requirement for NAD(P)H and lacks a conventional nicotinamide dinucleotide binding domain, it does appear that NAD(P)H has a biological role in reactivation of the enzyme (Teufel et al., 2015) by returning the flavin to the reduced resting state (∼0.100 s^–1^) from where a reaction with dioxygen can (re)form the flavin N5-oxide oxygenating intermediate. This proposal is supported by data that describes ∼36-fold lower binding affinity for NADPH compared to NADH while maintaining similar limiting rate constant for flavin reduction (∼0.100 s^–1^) (Teufel et al., 2015). Despite efforts, no structural evidence has been offered for the EncM ⋅NADPH complex (Teufel et al., 2015).

As mentioned above, conventional flavin monooxygenase chemistry utilizes a flavin-C4a-(hydro)peroxide (Entsch et al., 1976; Entsch and Ballou, 1989; Palfey and McDonald, 2010) to oxygenate target substrates. For EncM this intermediate was readily ruled out by the UV-Vis spectral characteristics. It was also observed that the generation of the EncM flavin oxygenating species from anaerobically reduced enzyme required dioxygen; anoxic chemical reoxidation resulted in oxidized inactive enzyme (Teufel et al., 2013). Additionally, when EncM that was reoxidized with ^18^O_2_ and reacted with a truncated racemic substrate, there was a 1:1 conversion to a diastereomeric mixture of products that were identified by NMR and MS as the ring-open derivatives of the predicted lactone (Teufel et al., 2013). These studies also revealed that the substrate-binding tunnel of EncM is specifically tailored to accommodate only the C7-(R)-enantiomer of the substrate, supporting the observed single configuration of the C4-hydroxyl in the enterocin product (Teufel et al., 2013). Based on these observations the EncM oxygenating species was proposed to be the flavin N5-oxide formed from a reaction of the reduced enzyme with dioxygen (Orf and Dolphin, 1974; Rastetter et al., 1979; Teufel et al., 2013). More direct evidence for the active N5-oxide flavin came in 2015 when researchers analyzed peptide fragments generated proteinase K digestion of EncM using LCMS and identified a mass consistent with the predicted His78-bound flavin N5-oxide fragment (Teufel et al., 2015). To definitively correlate the observed mass signal with the flavin-bonded oxygen molecule, chemically reduced EncM was reoxidized by ^18^O_2_ and ^16^O_2_ and then analyzed similarly by LCMS to confirm the generation of a masses consistent with a flavin N5 isotopic incorporation (Teufel et al., 2015).

**FIGURE 9:**
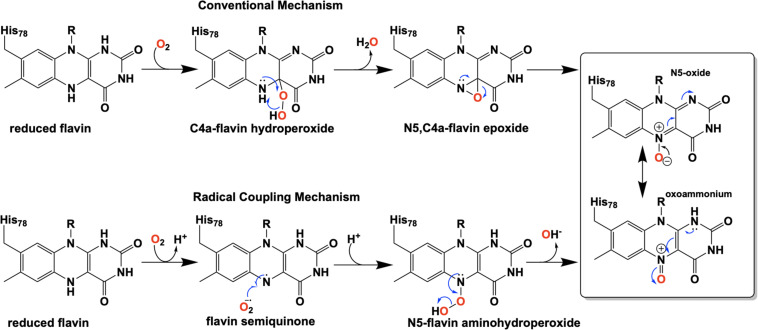
Proposed pathways for the formation of the flavin N5-oxide.

Extensive investigation into the mechanism and control of oxygen reactivity mediated by EncM has led to a better understanding of these unique enzymatic events (Teufel et al., 2015; Saleem-Batcha et al., 2018). Reaction of the reduced enzyme with dioxygen was originally proposed (Teufel et al., 2015) to involve the reduced flavin reacting conventionally with dioxygen resulting in a flavin-C4a-hydroperoxide that immediately undergoes water elimination to form an epoxide that is then opened to form the flavin N5-oxoammonium/N5-oxide (Figure 9, top). However, the orientation of the predicted C4a-peroxide would disfavor formation of the ring-strained oxaziridine and on this basis this mechanism has been replaced by a radical coupling mechanism (Scheme 9, bottom) (Teufel et al., 2015). This mechanism is defined by direct proton transfer between flavin N5 and dioxygen resulting in the formation of a protonated superoxide (peroxyl radical) and an anionic semiquinone which subsequently dehydrates to form the N5-oxoammonium/N5-oxo resonance pair. Stabilization of the flavin anionic semiquinone by EncM (Teufel et al., 2013) would promote the reaction with the protonated superoxide as a consequence of high spin density at the N5 (Massey, 1980; Barquera et al., 2003). Additionally, oxidation of reduced EncM by dioxygen occurred without the formation of detectable intermediates (Saleem-Batcha et al., 2018). These experiments also demonstrated that the observed rate constants titrate to a saturating dioxygen concentration and support a two-step mechanism with a binding event preceding adduct formation (Saleem-Batcha et al., 2018).

Direct structural insights for dioxygen reactivity were obtained from pressurized-dioxygen X-ray crystallography which exposed crystallized EncM to controlled pressures of oxygen. These structures displayed significant electron density proximal to the *re*-face of the isoalloxazine moiety and adjacent to the amphiphilic pocket previously identified (Teufel et al., 2013) not seen in either anaerobic or noble gas controls (Figure 8; Saleem-Batcha et al., 2018). A definitive oblong shape was observed in the omit map was proximal to three residues (V135, V136, and T139) within 3 Å of the observed density. These residues were assumed to be required for appropriate positioning for short range electron transfer to the flavin N5 (Saleem-Batcha et al., 2018). Curiously, many of the structures obtained in this study had no electron density indicative of flavinylation between H78 and the C8a of the isoalloxazine moiety of FAD as was observed in prior structures (Saleem-Batcha et al., 2018).

Mutational analysis of the residues that form the suspected dioxygen binding pocket largely destabilized the enzyme and mutation of adjacent residues (L144, L116, H138, and L117) resulted in diminished dioxygen occupancy of crystal structures and impeded catalysis and in one instance (T139V) oxidase activity was observed (Saleem-Batcha et al., 2018). It is likely this activity parallels previous observations of EncM inactivation, where atmospheric dioxygen aberrantly oxidizes the bound diketone to the triketone mimicking conventional turnover. This further supports a route of reactivation via flavin reduction and subsequent reaction with dioxygen to form the N5-oxide. Furthermore it also suggests that kinetic inactivation may be a function of truncated substrate analogs that do not fully preclude adventitious reaction by dioxygen.

**FIGURE 10:**
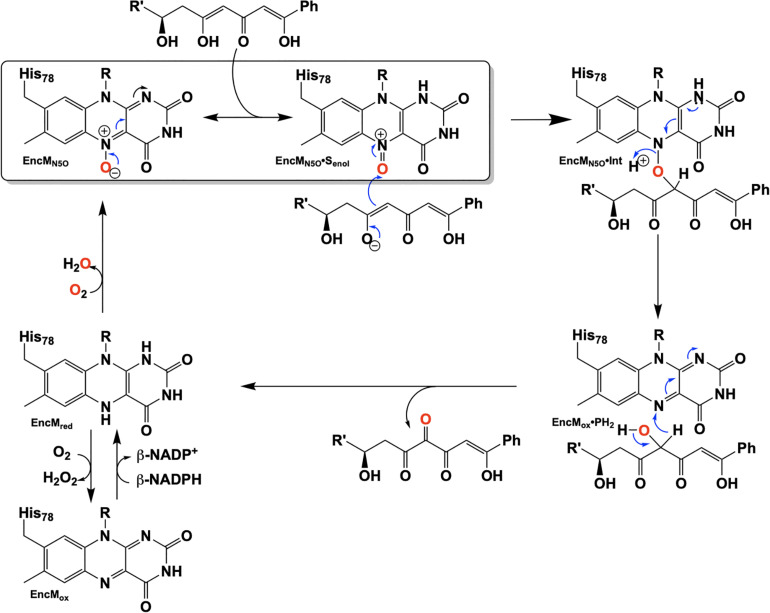
Hypothetical chemical mechanism of EncM.

Due to the intramolecular reactivity, preparation of the native substrate is difficult and analogs with truncated ketide chains have been used to study EncM (Teufel et al., 2013). The mechanism proposed from studies with these substrates is depicted in Figure 10. In this mechanism, the oxoammonium of the flavin undergoes attack by the proximal enolate of the substrate, resulting in the formation of amine-oxo-bridged species. This is cleaved by protonation yielding the flavin cofactor and generation of a C4-hydroxylated intermediate. Hydride transfer to the flavin produces the C4-ketone product and the reduced form of the flavin cofactor.

EncM presents the first example of a flavoprotein that requires a hyperoxidized flavin cofactor (flavin N5-oxide) for its catalytic function. This discovery prompts the question, given the similarity of the spectra of the flavin N5-oxide and two-electron oxidized flavin, is EncM an unusual flavoprotein that displays unique chemistry or is this just the first identified of a new class of enzymes?

## RutA

**FIGURE 11:**
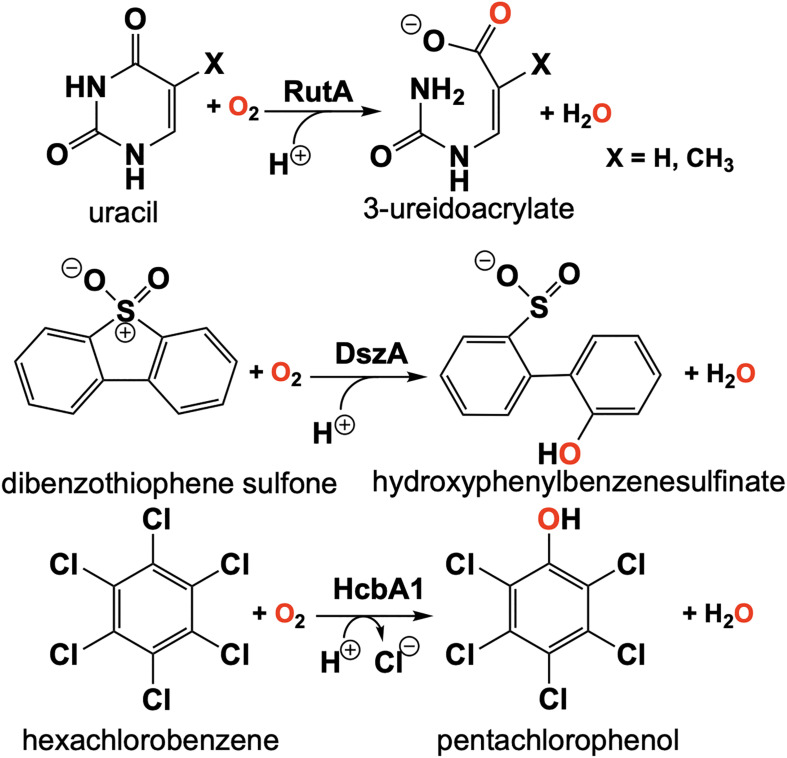
Reactions of N5-mediated monooxygenation enzymes; RutA, DszA, and HcbA1.

The discovery of the flavin N5-oxide, whose spectrum closely resembles that of two-electron oxidized flavin has prompted scrutiny of previous investigations of flavo-enzymes that exhibit unique oxygenation chemistries. The objective was to ascertain if the flavin N5-oxide is common or idiosyncratic. This inquiry has yielded the discovery a class of enzymes that catalyze the oxygenated cleavage of a diverse set of substrates by the enzymes RutA, DszA and HcbA1 that each utilize a flavin N5-oxide (Adak and Begley, 2016, 2017, 2019; Figure 11). It is of note that this newly described class of enzymes contains FMN and not an FAD as in the case of EncM. We will focus on the enzyme RutA as the hallmark example, which catalyzes the amide cleavage and oxygenation of uracil and thymine to form 3-ureidoacrylate or 2-methylureidoacrylate, respectively (Mukherjee et al., 2010).

It was the long standing notion that pyrimidines thymine and uracil were exclusively catabolized by one of two pathways described as oxidative or reductive (Vogels and Van der Drift, 1976). The reductive pathway is well described and is expressed in a diversity of organisms including archaea, bacteria and eukaryotes (Wasternack, 1978; Rawls, 2006; Piskur et al., 2007). This pathway follows NAD(P)H dependent catabolism of uracil or thymine to yield carbon dioxide, ammonia and either β-alanine (uracil) or β-aminoisobutyrate (thymine). The oxidative pathway is confined to a variety of bacteria and affords urea and either malonic acid (uracil) or methylmalonic acid (thymine) as products (Soong et al., 2001, 2002). However, Loh et al. (2006) described an additional pathway of pyrimidine catabolism encoded by the b1012 operon of *E coli* K-12. This novel third pathway relies on reducing equivalents from NAD(P)H to yield 3-hydroxypropionic acid (uracil) or 2-methyl-3-hydroxypropionic acid (thymine), carbon dioxide and ammonia (Loh et al., 2006; Figure 11). The discovery of RutA was a result of analysis of a strain of *E. coli* carrying the ntrB(Con) mutation that can utilize thymidine as the sole nitrogen source and lesions in the b1012 operon completely nullified this activity (Loh et al., 2006). These researchers also characterized the gene products of this operon as encoding a seven-enzyme system tentatively including, endoribonuclease (RutC), nitroreductase (RutE), flavin reductase (RutF), xanthine/uracil permease (RutG), putative isochorismatase (RutB), putative α/β hydrolase (RutD) and a monooxygenase (RutA).

**FIGURE 12:**
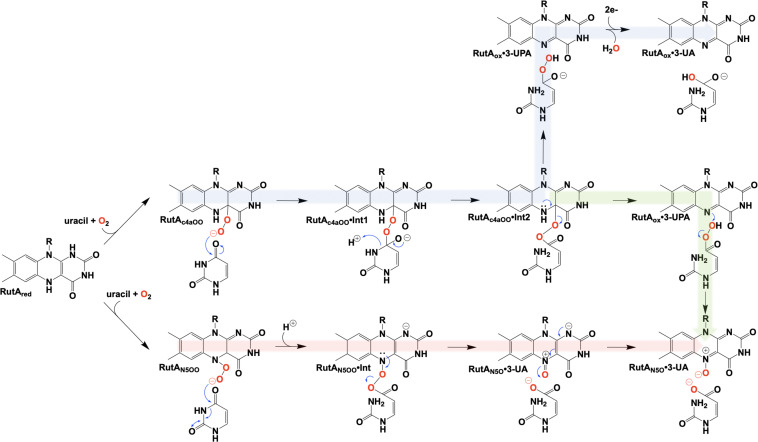
Proposed chemical mechanisms of RutA. Blue, initial mechanism utilizing a C4a-hydroperoxyl. Green, mechanism terminating in the formation of an N5-oxide complex. Red, direct oxygen transfer mechanism utilizing an N5-hydroperoxide and terminating with the formation of an N5-oxide complex.

**FIGURE 13:**
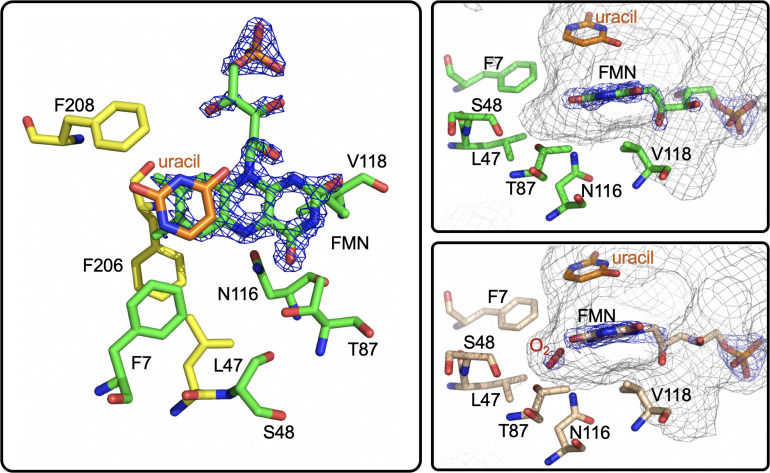
The active site of RutA. Left and right top, RutA in complex with uracil substrate (PDB code 5WAN). For both, active site residues that are involved in N5-oxo formation are shown in green. Right bottom, active site of RutA in complex with uracil under 15 bar dioxygen (PDB code 6TEG). Electron density (2fo-fc) is shown in blue mesh and the active site cavity is shown in gray mesh.

The initial step of the Rut pyrimidine catabolism pathway was originally described as a “oxidative hydrolysis” of uracil to yield 3-ureidoacrylate. It was quickly determined that this activity is catalyzed by RutA and RutF in the presence of FMN and NADH, where the presence of a flavin reductase (RutF) was required to reduce and transfer a flavin to RutA (Mukherjee et al., 2010). The formation of 3-ureidoacrylate was determined in these reactions by HPLC and cleavage of the N3-C4 bond was confirmed by ^13^C NMR in reactions utilizing ^13^C,^15^N-labeled uracil (Mukherjee et al., 2010). These results led researchers to propose a catalytic mechanism consistent flavin oxygenations that utilize a C4a-centered mechanism and a subsequent reduction reaction (Figure 12, blue). Initially it was unclear whether the acquisition of pyrimidine substrate was dependent on the presence or redox state of FMN. Only the Rut⋅Apyr⋅FMNH_2_ complex can undergo a reaction with dioxygen to transiently form the peroxyflavin species that facilitates the nucleophilic attack of the C4 of the pyrimidine resulting in ring cleavage. Though this mechanism is based on scientific precedent it does not describe the source of reducing equivalents for conversion of the predicted 3-ureidoacrylic peracid species. It was concluded that reduction must be either adventitious/non-catalytic or accomplished by a separate enzyme (Mukherjee et al., 2010). Furthermore, early crystal structures of RutA solved in the presence of uracil (PDB code 5WAN, 1.8 Å) (Figure 13) revealed an overall structure typical of group C FMN dependent flavin monooxygenases (Ellis, 2010) but did not contain an active site cysteine residue previously shown to stabilize the flavin C4a-hydroperoxide (Hall et al., 2009).

It is unclear specifically what led these researchers to revisit their initial mechanistic rationale, but 7 years subsequent to their initial proposal a new mechanism was offered (Adak and Begley, 2017; Figure 12, green). The mechanistic amendment was a result of heat quench experiments that reacted photo-reduced RutA with stoichiometric amounts of uracil under anaerobic conditions which when reacted with dioxygen resulted in the formation of a stabile flavin N5-oxide as determined by LC-MS and HPLC coelution with a synthesized flavin N5-oxide (Adak and Begley, 2017). Furthermore, a repeat of the aforementioned reaction carried out in the presence of ^18^O_2_ resulted in a 2 Da mass shift consistent with the incorporation of a single oxygen atom from molecular oxygen. Overall this mechanism parallels that of the original proposal where C4a oxygen activation catalyzes the cleavage of the 3,4-amide linkage of the pyrimidine, but terminates instead at a product elimination step that affords a flavin N5-oxide coupled to the cleavage of 3-ureidoacrylic peracid. Included is a second role for RutF, reinstating the reduced flavin in place of the N5-oxide.

Further insight into the catalytic mechanism of RutA came in 2020 when a group of researchers obtained crystal structures of oxidized RutA in the presence of non-convertible substrate analogs 4-thiouracil (PDB code 6SGM, 2.0 Å) and 2,4-dimethoxypryrimidine (PDB code 6SGN, 2.5 Å) (Matthews et al., 2020). These crystal structures confirmed the existence of a ligand binding pocket adjacent to the *si*-face of the isoalloxazine moiety (Figure 13; van Berkel et al., 2006; Mascotti et al., 2016). Surprisingly, very few side chain interactions exist between the bound pyrimidine and RutA despite prior evidence that flavin N5 adduct formation is predicated on substrate association (Adak and Begley, 2017). Furthermore, ligand association did not result in any obvious changes to the protein scaffold. These structures also revealed the presence of a second, more confined (∼24 Å^3^) active site cavity lined with non-polar side chains (L65, V136, and F224) proximal the substrate binding pocket that is necessary to bind the hydrophobic dioxygen molecule (Figure 13, gray mesh). The distal portion of the presumed dioxygen binding pocket is occupied by the polar side chains of T105 and N134 and likely stabilizes the hydrophilic protonated superoxide through hydrogen bonding. Mutational analysis of residues T105, N134, and V136 that line the putative dioxygen binding cavity abolished activity except in the case of T105S which retained ∼80% of WT activity (Matthews et al., 2020). The absolute requirement of the positioning and identity of amino acid residues involved coupled with the confined nature of active cavity (radius ∼1.8 Å compared to the anisotropic Van der Waals radii of gaseous dioxygen of 1.77) highlights that proper dioxygen positioning is fundamental to catalysis (Matthews et al., 2020).

Aside from traditional C4a-flavin oxygenase enzymes, dioxygen reactivity of flavoproteins is most commonly bimolecular and does not involve a prior complex (McDonald et al., 2011; Chaiyen et al., 2012; Huijbers et al., 2014; Saleem-Batcha et al., 2018). To confirm the existence of an *bona fide* oxygen binding pocket, O_2_-pressurized X-ray crystallography was used to expose crystals to varying pressures of dioxygen from 0 to 15 bar immediately preceding freezing in liquid nitrogen (Matthews et al., 2020). Significant electron densities not seen in anaerobic controls was observed adjacent to the *si*-face of the flavin in all crystals exposed to dioxygen prior to diffraction and the intensity of the electron density titrated with increased oxygen pressures of 0 bar (PDB code 6TEE, 2.2 Å) 5 bar (PDB code 6TEF, 1.8 Å) and 15 bar (PDB code 6SGG, 1.8 Å). Modeled dioxygen conforms well to the observed electron densities generated from 2fo-fc maps (PDB code 6TEG, 1.9 Å) (Figure 13). The protein therefore directs the proximal oxygen atom to localize ∼2.1 Å from the flavin N5, indicative of a direct interaction with this position and significantly closer than the distance to the flavin-C4a (3.1 Å). Furthermore, the angle between the modeled dioxygen the flavin N5 is 99°, while the angle to the flavin-C4a position is 118°, predicting the former as the preferred point of reaction.

In 2020, Matthews et al. added another mechanistic addendum to the proposed mechanism of RutA (Figure 12, red) (Matthews et al., 2020). The mechanism proposed initiates with the formation of the flavin N5-oxide generated from the RutA⋅FMNH_2_ complex via a radical coupling mechanism as described for EncM (Figure 9). This is a deviation from the initially proposed mechanisms that form such a species concomitant with product release (Adak and Begley, 2017). This modification is supported by HPLC and LCMS analysis that confirm the formation of flavin N5-oxide in reactions carried out in the presence of both convertible and non-convertible substrate analogs (2-thiouracil, 4-thiouracil, 2,4-dithiouracil, DMP, cytosine, and 1,3-cyclohexanedione) (Matthews et al., 2020). Despite the formation of active flavin N5-oxide with all analogs tested, HPLC analysis of these reactions confirmed only 2-thiouracil was able to illicit catalysis (Matthews et al., 2020) and confirmed the absolute requirement of the presence of a pyrimidine substrate with an intact amide moiety for turnover. Establishment of this initial oxygenating species would then predict that the actual identity of the oxygenating species is the flavin N5-peroxide and not the flavin-C4a-peroxide (Figure 12). As such, the neutral N5-peroxide would act as a soft nucleophile promoting an umbrella/nitrogen inversion abridging the distance to the substrate amide for cleavage and subsequent direct/covalent oxygenation forming 3-UA and the flavin N5-oxide.

The discovery of a third flavin oxygenating species unique from flavin-C4a and recently discovered flavin N5-oxide, provides for a redox neutral oxygenative cleavage. This discovery convincingly describes the existence of a new oxygenating class of flavoproteins, unique in both mechanism and scaffold, that utilize the electron donating properties of the hyperoxidized isoalloxazine to accomplish a unique net transfer of a hydroxide. To date three examples of such chemistry have been described and provide an identifiable motif that is found in a number of oxygenase enzyme primary sequences (Matthews et al., 2020). These results, in part, suggest that reinterpretation of previously described group C monooxygenases could provide additional examples of N5 oxygenative chemistries that have been dogmatically assigned as C4a centered oxygenation.

## Conclusive Remarks

**FIGURE 14:**
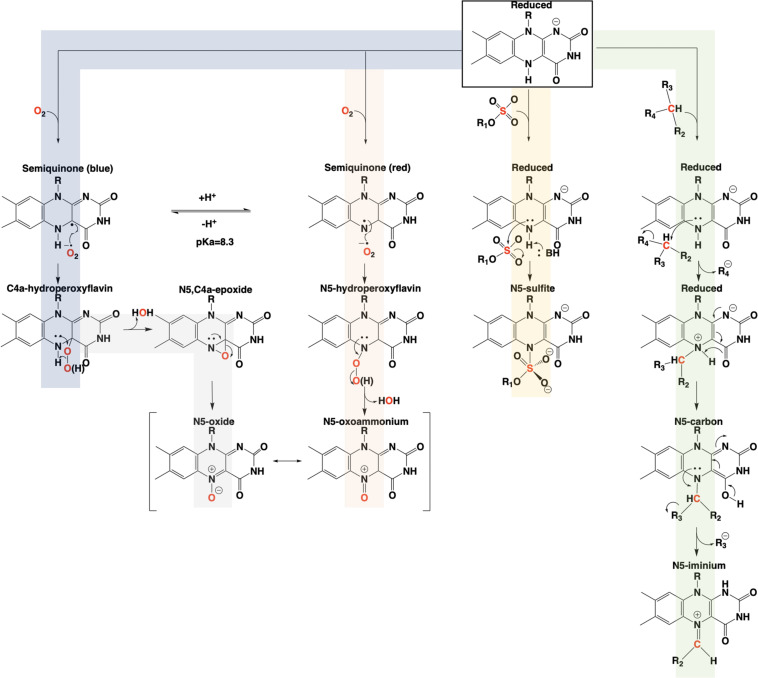
Summary of the proposed pathways for covalent catalysis of reduced flavin in enzymes.

The involvement of the flavin N5 position in enzymatic reactions has become a focus for flavoenzyme chemistry in recent years (Piano et al., 2017; Romero et al., 2018; Leys and Scrutton, 2020). With the exception of long-range electron transfer (Schnackerz et al., 2004) and flavinylation (Sharma et al., 2018), the N5 position is the point of entry for electrons from substrate molecules to form the reduced flavin. The role of the reduced flavin as a nucleophile in the formation of adducts at N5 is now well-supported (Marshall et al., 2017; Sobrado and Tanner, 2017; Leys, 2018). Though outside the scope of this review, there are examples of N5 adducts that arise with reduction of the flavin such as ADPS (Nenci et al., 2012; Figure 1). Many of the recently discovered enzymes purported to utilize the N5 position of the isoalloxazine have proposed chemical mechanisms, for which key details remain unsettled (Xiang et al., 2004; Teufel et al., 2015; Adak and Begley, 2017); this is particularly true for oxygenase chemistry. Nonetheless, in the reduced state of the flavin broad reactivity propensities can be identified for the C4a and N5 positions. The flavin-C4a position is more often involved in one-electron chemistry; commonly the reductive activation of molecular oxygen and formation of C4a-peroxo adducts or hydrogen peroxide (Massey, 1994). Conversely, the N5 position is more often the site for two-electron reactivity forming covalent intermediates. However, the intermediacy of semiquinone forms that have high spin density at the C4a (blue semiquinone) or N5 (red semiquinone) suggests that one-electron reactivity is possible at either center, and this remains a source for mechanistic conjecture (Teufel et al., 2015; Dai et al., 2018; Matthews et al., 2020). Figure 14 summarizes the proposed chemistries of reduced flavins with respect to the C4a and N5 positions and includes hypothetical pathways for N5 oxygenation.

In conclusion, the adjacent C4a and N5 positions of the reduced isoalloxazine provide broad and tunable capabilities to flavin-dependent enzymes. The versatility of these centers is most evident in the fact that they cannot currently be definitively predicted from sequence and/or structure to perform a specific type of chemistry (Teufel et al., 2013). It would appear that C4a and N5 each have dominant chemical propensities in enzymes but, each to some extent can be adapted to functions that are more commonly attributed the other.

## Author Contributions

BB was responsible for the preparation of the text and figures of this manuscript. GM was responsible primarily for editing and proofreading. Both authors contributed to the article and approved the submitted version.

## Conflict of Interest

The authors declare that the research was conducted in the absence of any commercial or financial relationships that could be construed as a potential conflict of interest.
